# The pROS of Autophagy in Neuronal Health

**DOI:** 10.1016/j.jmb.2020.01.020

**Published:** 2020-04-03

**Authors:** Lucia Sedlackova, George Kelly, Viktor I. Korolchuk

**Affiliations:** Institute for Cell and Molecular Biosciences, Newcastle University, Campus for Ageing and Vitality, Newcastle Upon Tyne, NE4 5PL, UK

**Keywords:** autophagy, ROS, neurodegeneration, oxidation, cysteine modification

## Abstract

Autophagy refers to a set of catabolic pathways that together facilitate degradation of superfluous, damaged and toxic cellular components. The most studied type of autophagy, called macroautophagy, involves membrane mobilisation, cargo engulfment and trafficking of the newly formed autophagic vesicle to the recycling organelle, the lysosome. Macroautophagy responds to a variety of intra- and extra-cellular stress conditions including, but not limited to, pathogen intrusion, oxygen or nutrient starvation, proteotoxic and organelle stress, and elevation of reactive oxygen species (ROS). ROS are highly reactive oxygen molecules that can interact with cellular macromolecules (proteins, lipids, nucleic acids) to either modify their activity or, when released in excess, inflict irreversible damage. Although increased ROS release has long been recognised for its involvement in macroautophagy activation, the underlying mechanisms and the wider impact of ROS-mediated macroautophagy stimulation remain incompletely understood.

We therefore discuss the growing body of evidence that describes the variety of mechanisms modulated by ROS that trigger cytoprotective detoxification via macroautophagy. We outline the role of ROS in signalling upstream of autophagy initiation, by increased gene expression and post-translational modifications of transcription factors, and in the formation and nucleation of autophagic vesicles by cysteine modification of conserved autophagy proteins including ATG4B, ATG7 and ATG3. Furthermore, we review the effect of ROS on selective forms of macroautophagy, specifically on cargo recognition by autophagy receptor proteins p62 and NBR1 (neighbour of BRCA1) and the recycling of mitochondria (mitophagy), and peroxisomes (pexophagy). Finally, we highlight both, the standalone and mutual contributions of abnormal ROS signalling and macroautophagy to the development and progression of neurodegenerative diseases.

## Highlights

• ROS are messengers that modify protein activity by PTMs.
• ROS-mediated PTMs regulate activity and specificity of autophagy proteins.
• Increase in autophagy mediates rapid clearance of oxidised cargo and ROS sources.
• The importance of ROS-mediated autophagy is highlighted in neurodegeneration.

## Graphical abstract

**Figure:**
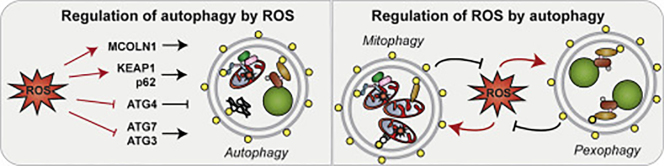

## Macroautophagy: A Brief Overview

Macroautophagy, from herein referred to as autophagy, refers to the dynamic rearrangement of cellular membranes to engulf cytoplasmic cargo in a double-membraned compartment (autophagosome) and its delivery to the lysosome. Autophagy is the principal degradation system for long-lived proteins and the only known pathway of whole organelle recycling [1]. The process is regulated by a family of core autophagy (ATG) proteins that are largely conserved from yeast to mammals. In higher organisms, the core members associate with their regulators into five functional complexes that initiate autophagosome formation, cargo docking, vesicle expansion and closure [1]. Briefly, activation of the first complex, the unc-51-like autophagy activating kinase 1 (ULK1) protein kinase complex, initiates the formation of an isolation membrane, which is followed by membrane elongation mediated by delivery of vesicles containing ATG9 [1,2]. Phosphatidylinositol 3-phosphate (PI(3)P), a product of the class III PI(3)-kinase complex, then serves to recruit the last two autophagy complexes containing ATG12 and a member of a family of mammalian ATG8 orthologues (microtubule associated protein 1 light-chain 3 proteins (MAP1LC3A-C/LC3A-C), the γ-aminobutyric acid receptor–associated protein (GABARAP), and the γ-aminobutyric acid receptor–associated protein-like 1 and 2 (GAPARAPL 1/2)) [3], that undergo ubiquitin-like conjugation and assist with cargo docking, vesicle formation and degradation [4]. Of the two known forms of autophagy, basal and induced, we explore the role of reactive oxygen species (ROS) in the initiation and regulation of the latter form aimed at cytosol detoxification and its relevance to the health of the neuronal systems.

## Reactive Oxygen Species: The Types, Sources and Cellular Functions

ROS is a collective term used to describe species formed as intermediates of dioxygen (O_2_) reduction to H_2_O. In its ground state, the O_2_ molecule harbours two unpaired valence electrons and is thus considered a radical species [5]. However, due to the parallel spin of its valence electrons and the nonradical nature of most elements, O_2_ is unlikely to participate in biological reactions without first overcoming the spin restriction [5]. In aerobically adapted cells, oxygen activation is achieved by its bonding to transition metals that are capable of overcoming oxygen's spin restriction by mediating a series of one-electron reduction reactions via a sequence of intermediates [6]. The partially reduced O_2_ intermediates include the superoxide anion (O_2_^•−^) that contains a single unpaired electron, the hydrogen peroxide (H_2_O_2_) that contains no unpaired electrons, and the hydroxyl radical (OH^•^) that contains a single unpaired electron [6]. An unpaired electron on the outer orbital of O_2_^•−^ and OH^•^ increases their ability to take part in one-electron oxidative transfer reactions leading to macromolecule modification and, if excessive, damage and loss of function.

Once thought to only form in large quantities on ionising radiation, it is now recognised that ROS escape complete reduction in biologically significant quantities. ROS toxicity is intensified by O_2_^•−^ interaction with nitrogen monoxide (nitric oxide, NO^•^), another radical species produced by nitric oxide synthase (NOS) enzymes as a signalling molecule important in vasodilation, neurotransmission and synaptic plasticity [7]. Peroxynitrite anion (ONOO^−^), a product of NO^•^ oxidation by O_2_^•−^, is a reactive nitrogen species (RNS) that is capable of altering cellular signalling by protein modification via formation of irreversible nitrosyl adducts on tyrosine residues or reversible oxidative modification of cysteine, methionine and tryptophan residues [7]. In addition, peroxynitrite can damage macromolecules including nucleic acids, proteins and lipids and initiate various forms of cell death [8]. Although largely regarded as detrimental to cellular health, ROS have been rebranded from villains to signal messengers that, in the right dose, rejuvenate cells and increase health span and longevity [9].

## Redox Regulation of Autophagy

The balance between beneficial and deleterious roles of ROS is heavily dependent on the efficiency of cellular detoxification systems. Cellular redox homeostasis is maintained by a defence system of endogenous enzymatic (e.g. superoxide dismutases (SODs), catalase, glutathione peroxidase (GPx), glutathione reductase (GRx), thioredoxins and peroxiredoxins) and nonenzymatic ROS scavengers (e.g. glutathione (GSH), coenzyme Q, vitamins C and E) [10]. This first line of defence scavenges and detoxifies ROS and thus prevents the propagation of ROS signalling. However, large bursts of localised ROS can overwhelm the endogenous antioxidant systems and promote ROS signalling, or when in excess, confer oxidative damage on cellular macromolecules. The second line of defence comprises systems that remove and recycle oxidised cytotoxic macromolecules [11]. Cells rely primarily on the proteasome- and autophagy-mediated clearance of oxidised cargo. Studies focussing on the role of the proteasome in oxidised protein recycling reveal a link between oxidation and increased activity of the proteasome [12]. ROS-mediated dissociation of the 26S proteasome into its 19S (regulatory complex) and 20S (catalytic core) components removes the 20S-mediated specificity for ubiquitylated substrates and thus promotes rapid recycling of oxidised proteins [12,13]. In addition, exposure to elevated ROS was also linked to increased proteasome synthesis [14,15]. However, the limited functionality of the proteasome capable of degrading primarily short-lived nonaggregated proteins suggests that the main catabolic pathway that partakes in cellular detoxification by sequestration and clearance of oxidised cargo is autophagy.

Similar to the response of the proteasome, autophagy stimulation by ROS was observed on cell treatment with H_2_O_2_ and nutrient starvation experiments, in which increase in H_2_O_2_ release correlated with autophagosome formation [16]. A later study by Gibson group demonstrated that prolonged withdrawal of glucose, l-glutamine, pyruvate and serum or all amino acids and serum, led to increased release of O_2_^•−^, while amino acid starvation also increased cellular levels of H_2_O_2_ [17]. Importantly, increased stimulation of autophagy flux correlated with O_2_^•−^, but not with H_2_O_2_ species. An unexpected finding of the study revealed that addition of exogenous H_2_O_2_, used in multiple *in vitro* studies to mimic endogenous ROS release, stimulated O_2_^•−^ release that in turn initiated autophagosome formation. Altogether, these findings support the notion that endogenous ROS release and exogenous H_2_O_2_ supplementation are sufficient drivers of the autophagy pathway. Here, we explore the mechanisms by which proteins sense increased levels of cellular oxidative stress, upregulate levels of autophagy components, enhance autophagy efficiency and altogether recycle not only the cellular macromolecules damaged by ROS, but also the organelles that release ROS in the first place (Table 1).

**Table 1:** Redox-sensitive proteins in autophagy.

Protein	Function in autophagy machinery	Redox-Sensitive Cys residues	Outcome of Oxidation	Autophagy Outcome	
MCOLN1	Indirect	not identified	Ca^2+^ release, TFEB activation [23]	↑	ATG and lysosomal gene transcription
KEAP1	Indirect	multiple	NRF2 release [53]	↑	ATG, NRF2, p62 transcription
p62	Selective cargo recognition	Cys^105^ and Cys^113^	Oligomerisation [47]	↑	Autophagy stimulation
ATG3	Autophagosome elongation	Cys^264^	Loss of LC3-PE formation [37]	↓	Autophagosome formation
ATG4B	Pro-LC3 processing, LC3-PE deconjugation	Cys^74^ or Cys^78^	Loss of deconjugating activity [16]	↑	Autophagosome formation
ATG7	Autophagosome elongation	Cys^572^	Loss of LC3-PE formation [37]	↓	Autophagosome formation
ATM	Indirect	Cys^2991^	ATM recruitment to peroxisome [80]	↑	Pexophagy

### Transcriptional regulation of autophagy

Transcriptional upregulation of key autophagy genes in response to various nutritional stresses depends on several transcription factors (TFs) including transcription factor EB (TFEB), forkhead box O (FoxO), early growth response (EGR-1), E2F transcription 1 (E2F1), Jun and farnesoid X receptor (FXR) (summarised in Ref. [18]). It is currently not known whether direct interaction with ROS or oxidative post-translational modifications (PTMs) have an effect on the activity or localisation of most of these factors, but some direct and indirect links between ROS and upstream regulators of TF activity or localisation have been described.

**Figure 1:**
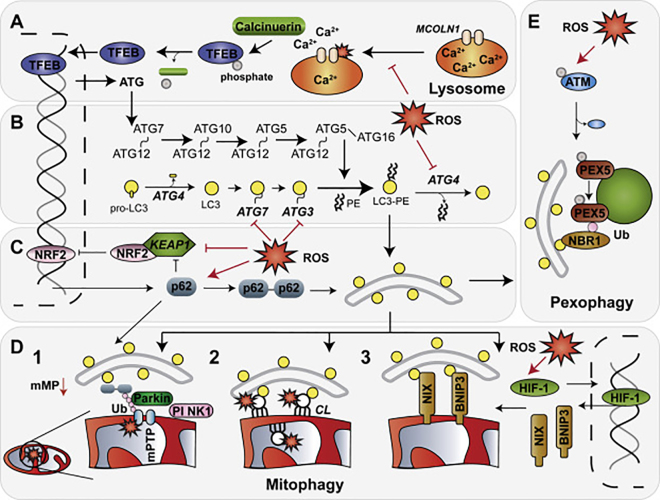
**Autophagy regulation by ROS**. Increased presence of ROS affects autophagy mechanisms via modification of multiple proteins involved in autophagy regulation. (A) Oxidative modification of MCOLN1 releases lysosomal Ca^2+^ stores that via calcineurin phosphatase activity, promote TFEB nuclear localisation and transcriptional activity of lysosomal and *ATG* genes. (B) ROS has a dual role in regulating autophagosome formation. First, oxidation of residues near the catalytic core of ATG4 reportedly inhibits its deconjugating activity and results in increased autophagic flux. Second, ATG7 and ATG3 activity is inhibited by oxidation-dependent ATG7-ATG3 heterodimer formation on LC3 depletion. (C) KEAP1-NRF2 heterodimer is disrupted either by KEAP1 oxidative modification or by increased p62 binding. Liberated NRF2 that escapes degradation and cytoplasmic sequestration initiates a positive feedback loop of NRF2 and p62 expression. Intermolecular cysteine bond formation of p62 also stimulates autophagy flux. (D, E) Selective recycling of mitochondria (mitophagy) and peroxisomes (pexophagy) is enhanced by ROS. (D) Transient bursts of ROS trigger mPTP opening, localised depolarisation and PINK1 stabilisation at the OMM (D1). Mitophagy is initiated by Parkin recruitment, OMM protein ubiquitylation (Ub) and recognition by autophagy receptor proteins. Mitochondrial lipid peroxidation triggers CL externalisation and LC3 docking (D2). Elevated intracellular ROS levels promote stabilisation and transcriptional activity of HIF-1 (D3). Increased levels of BNIP3 and NIX dock on OMM and promote mitochondrial recycling by directly interacting with LC3. Selective labelling of oxidised or superfluous organelles triggers mitophagy. (E) ROS-activated ATM localisation to peroxisomes results in PEX5 phosphorylation, ubiquitylation and autophagy receptor recruitment. Selective autophagy of ROS-producing organelles decreases intracellular ROS levels and restores homeostasis.

TFEB belongs to a microphthalmia family of bHLH-LZ transcription factors (Mit/TFE) that play a crucial role in autophagy stimulation and lysosomal biogenesis [19]. TFEB function as a master regulator of lysosomal biogenesis that occurs via positive stimulation of the coordinated lysosomal expression and regulation (CLEAR) network of genes [20]. In addition to regulation of lysosome biogenesis, an *in vitro* TFEB overexpression (OE) study identified multiple autophagy genes that were consistently and significantly upregulated in various OE models including ATG4D, ATG9B, LC3B and SQSTM1/p62 [21]. A link between TFEB activity and intracellular ROS was reported from models of starvation [22] or exogenous and endogenous increase in ROS levels [23]. In both studies, authors observed activation of an indiscriminate lysosomal cation channel, mucolipin 1 (MCOLN1/TRPML1) that led to Ca^2+^ influx into the cytoplasm and stimulated calcineurin phosphatase that dephosphorylates TFEB, thus leading to its translocation into the nucleus followed by increased autophagy flux (Fig. 1A) [22,23]. Moreover, authors of a recently published study demonstrated that TFEB and other members of the Mit/TFE family (transcription factor E3 (TFE3) and microphthalmia-associated transcription factor (MITF)) are sensitive to ROS-mediated activation [24]. In this study, oxidation of the only Cys residue in TFEB, Cys^212^, led to loss of TFEB phosphorylation, its rapid (within 8 min) translocation to the nucleus and increased TFEB/TFE3/MITF-dependent transcription of the CLEAR genes, some autophagy genes and, interestingly, *MCOLN1* [24].

At the same time, evidence of a clear link between ROS and activation of FoxO, Jun, EGR-1, E2F1 and FXR TFs, all of which regulate transcription of multiple autophagy genes [18], remains limited. A link between ROS and FoxO family members focuses on the modulation of FoxO activity and localisation due to a) phosphorylation events of upstream receptor tyrosine kinases; b) acetylation status regulated by upstream acetyltransferases and deacetylases; and c) FoxO ubiquitylation (reviewed in Refs. [25,26]). The strongest link between cellular redox status, FoxO activation and autophagy stimulation comes from studies of sirtuin (SIRT)-mediated deacetylation of FoxO1 and FoxO3 isoforms that are well established as inducers of autophagy [26]. Although SIRT1 and SIRT3 were shown to activate FoxO1 and FoxO3 in response to various stresses [[27], [28], [29], [30]], and both SIRTs are indirectly activated by ROS, unequivocal evidence of a ROS-SIRT-FoxO-autophagy axis is not yet available. Similarly, gene expression, protein levels and nuclear localisation of the EGR-1 transcription factor all increase on cell stimulation with H_2_O_2_ in a JNK (c-Jun NH_2_ terminal kinase)- and ERK (the extracellular signal-regulated kinases)-dependent manner [31]. Importantly, stimulation of EGR-1 activity by ionising radiation was shown to increase expression of the ATG4B protein and initiate prosurvival autophagy [32]. Finally, there is currently no indication of ROS-mediated regulation of either E2F1 or FXR transcription activity.

In summary, multiple transcription factors are involved in autophagy regulation during adaptation to a variety of cellular stresses. The extent and *in vivo* relevance of ROS-mediated stimulation of signalling cascades upstream of TF activation is not known and requires further enquiry. The strongest link between ROS and TF activation has so far been demonstrated in TFEB activation. Direct oxidation of TFEB Cys^212^ and indirect MCOLN1-Ca^2+^-calcineurin cascade-mediated TFEB dephosphorylation promotes its translocation to the nucleus that leads to increased lysosome biogenesis and expression of several core autophagy proteins involved in autophagosome expansion (ATG4B, ATG9B, LC3B), autophagosome maturation (LC3B) and selective cargo recognition (p62) [22,23].

### Regulation of autophagy initiation

Synthesis of a membrane-bound form of ATG8 on nascent and maturing autophagosomes is crucial for autophagy progression and cargo recruitment [33]. Attachment of ATG8 proteins to autophagosome membranes is achieved by a covalent bond linkage between ATG8 and phosphatidylethanolamine (PE). Formation of the ATG8-PE conjugate is assisted by three classes of enzymes that sequentially activate (E1-like enzyme), conjugate (E2-like enzyme) and ligate (E3-like enzyme) two substrates in a manner similar to the ubiquitylation pathway [34]. Formation of ATG8-PE is first potentiated by a pro-ATG8 C-terminal processing by ATG4 (A-D) and then mediated by the conjugation system consisting of ATG7 (E1), ATG3 (E2) and the ATG5-ATG12 conjugate (E3) [1,35]. Similarly, the ATG5-ATG12 conjugate formation is assisted by ATG7 (E1) and ATG10 (E2) enzymes in an E3-independent process [36] (Fig. 1B).

Evidence first reported in 2007 and followed up by the most recent report in 2018 suggests that several members of core autophagy machinery are regulated by cellular redox state by disulphide bond formation and oxidative amino acid residue modification [16,37]. First, members of the ATG4 family function as cysteine (Cys)-dependent proteases and mediate the initial step of ATG8 conjugation to PE by exposing a conserved C-terminal glycine residue of ATG8 by proteolytic cleavage of the downstream C-terminal region [38]. Moreover, ATG4 also acts to hydrolyse LC3-PE conjugates from the nascent autophagosome that is crucial for correct substrate localisation into autophagosomal lumen. Studies of yeast and human ATG4 homologues involved in ATG8 processing report that the catalytic activity of ATG4 is suppressed by oxidative stress. First reported from human cells, the deconjugating activity, but not the LC3 processing activity of ATG4B is rapidly inactivated by a dithiothreitol (DTT)-sensitive oxidative modification of either the catalytic (Cys^74^) or a neighbouring (Cys^78^) cysteine residue [16]. Evidence from yeast indicates that formation of a disulphide bond between noncatalytic residues (Cys^338^ and Cys^394^) suppresses Atg4 activity [39]. Interestingly, both studies report that oxidative inhibition of ATG4 enzymatic activity increases autophagosome formation, although the underlying mechanism of this effect is not fully understood.

Similarly, direct oxidation of catalytic Cys residues was recently reported to play a role in the inhibition of ATG7 and ATG3 activity [37]. The E1-like (ATG7) and E2-like (ATG3) enzymes of the LC3-PE and ATG5-ATG12 conjugation systems were found to associate with LC3A and LC3B by the formation of a reversible thioester bond that was lost after autophagy induction by amino acid starvation [37]. LC3-free ATG7 and ATG3 were then prone to H_2_O_2_-mediated redox regulation by the formation of an intermolecular disulphide bond between the ATG3 and ATG7 catalytic thiols (Cys^264^ and Cys^572^, respectively) that led to impaired LC3-PE conjugation and the loss of autophagy flux. Interestingly, owing to the shielding of their cysteine residues, the LC3-ATG7 and LC3-ATG3 complexes were resistant to oxidation-mediated ATG7-ATG3 disulphide dimer formation [37].

Collectively, these data suggest a mechanism by which oxidation of the core autophagy machinery increases mature autophagosome formation by inhibiting the hydrolysing activity of ATG4B [16]. In addition, recent evidence suggests a self-regulatory mechanism by which the ATG8/LC3-PE formation and increased autophagy flux mediated by ROS are inhibited by ATG3-ATG7 disulphide heterodimer formation on depletion of intracellular ATG8/LC3 pools [37]. In healthy, nonstarved cells, this mechanism would lead to a regulated and rapid clearance of oxidised cargo. However, it is becoming increasingly clear that chronic exposure to ROS or concurrent starvation could derail this system and lead to autophagy impairment due to loss of ATG8/LC3-PE conjugation.

### Regulation of cargo recognition

Recruitment of the autophagic machinery to its cargo is mediated by autophagy receptor proteins [33]. Autophagy receptors are proteins that fulfil three requirements: direct interaction with LC3 via an LC3-interacting region (LIR), substrate binding (i.e. through a ubiquitin binding domain), and an inherent ability to polymerise or aggregate [33]. The first discovered and best-characterised selective autophagy receptor, p62 (also known as sequestosome 1 (SQSTM1)), was found to participate in the degradation of bacterial and viral pathogens, protein aggregates, mitochondria, peroxisomes and secretory granules (summarised in Ref. [33]). High-order structure formation of p62 (i.e. multimer, oligomer, aggregate) is key to its function as an autophagy receptor [40]. p62 contains several functional domains interlinked by unstructured regions. Of the structured domains, the N-terminal PB1 (Phox and Bem1p), ZZ (ZZ-type zinc finger), and the C-terminal UBA (ubiquitin associated) [41,42] domains feature in the context-dependent p62 high-molecular weight species formation and phase separation. For example, PB1 domain of p62 promotes its oligomerisation via electrostatic PB1-PB1 interactions and facilitates cargo condensation before enclosure within an autophagosome [43].

By contrast, an unstructured region between the PB1 and ZZ domains (also referred to as a regulatory linker region or an electrostatic PB1 bridge [44,45]), and the ZZ domain itself, contain cysteine residues that can contribute to p62 oligomerisation mediated by the covalent disulphide bond linkage [46,47]. Intermolecular disulphide bond formation was found to be crucial for p62 aggregation and p62-LC3 interaction in response to Nt-Arg (N-terminal arginine of arginylated substrates) binding to the ZZ domain [46,48,49]. The authors of these studies hypothesised that it is the conformational change triggered by Nt-Arg binding that induces p62 oligomerisation which facilitates further interactions with autophagy machinery through its PB1 and LIR domains. The formation of disulphide bonds observed in p62 upon Nt-Arg binding to the ZZ domain [46] points towards the role of ROS in the process of Cys oxidation and p62 oligomerisation in response to Nt-Arg, although the source of these ROS had not been investigated. Interestingly, one cysteine residue in p62 that appears to be involved in the formation of intermolecular disulphide bonds in response to Nt-Arg is Cys^113^ located in the regulatory linker region [46]. Although the identity of other cysteine residue(s) required for the formation of p62 chains remains unknown, Cys^113^ together with Cys^105^ has been implicated in the ability of p62 to sense cellular redox status. Specifically, in response to elevated ROS levels redox-sensitive cysteine residues Cys^105^ and Cys^113^ have been suggested to form intermolecular disulphide bonds, assist p62 oligomer assembly and activation of prosurvival autophagy in response to oxidative stress [47,50]. Altogether, the ability of p62 to become oxidised and form disulphide bonds appears to be essential for the upregulation of autophagy in response to proteotoxic or oxidative stress.

In addition to its structure and multimer formation, oxidative stress regulates p62 protein levels via its transcriptional regulation by the nuclear factor erythroid 2-related factor (NRF2), a master regulator of the cellular antioxidant response (Fig. 1C). In basal conditions, NRF2 associates with a homodimer of its binding partner, Kelch-like ECH-associated protein 1 (KEAP1) [51,52], and undergoes rapid ubiquitylation and proteasomal degradation. Elevation of intracellular ROS levels leads to oxidation of Cys residues and subsequent disulphide bond formation and conformational changes in KEAP1 that ultimately result in the release of NRF2 and its translocation to the nucleus [53]. Once inside the nucleus, NRF2 initiates transcription of genes that contain an antioxidant response element (ARE) in their promoter [54]. Among the over 600 genes that are targets of NRF2 are several core ATG proteins, selective autophagy receptors and, importantly, NRF2 and p62 themselves [[54], [55], [56]]. Furthermore, via its interaction with KEAP1, p62 disrupts one of KEAP1 binding sites with NRF2 and thus inhibits NRF2 degradation [55,57,58]. Therefore, elevation of intracellular oxidative stress leads to the establishment of a p62-KEAP1-NRF2-ARE feed-forward loop that, among other pathways, stimulates autophagy and leads to detoxification of the damaged cytoplasmic contents (Fig. 1C). This loop is broken on ROS detoxification, p62 recycling and NRF2-KEAP1 heterodimer formation.

Finally, owing to the similarity of p62 to another selective autophagy receptor, the neighbour of BRCA1 (NBR1) [33], it is highly likely that the redox-sensitivity may not be unique to p62. Indeed, data from Komatsu group demonstrates that NBR1 is transcriptionally upregulated in response to treatment with a strong oxidant, sodium arsenite [59]. NBR1 has so far been identified for its involvement in selective autophagy pathways that degrade protein aggregates and peroxisomes [33] and it will be interesting to see whether cellular ROS can regulate these processes via direct NBR1 oxidation.

### Regulation of selective organelle degradation

In healthy cells, most intracellular endogenous ROS are released as by-products of energy generation from macronutrients including glucose (in mitochondria) and fatty acids (in mitochondria and peroxisomes). At low levels, ROS act as messengers that communicate the metabolic status to cytoplasmic components and regulate cellular adaptation to metabolic stresses [60]. However, dysfunction of either organelle can lead to pathological ROS production that can overwhelm organellar and cytoplasmic antioxidant defences. It could be hypothesised that in this case, oxidative modification of redox-sensitive sentinels may promote recruitment of autophagic machinery to the site of damage to catabolise the source of ROS and protect the cell from further damage.

Mitophagy refers to the selective degradation of mitochondria, a network of double-membraned organelles that act as hubs of cellular energy production, signalling and viability [61]. Three distinct pathways can mediate ROS-induced mitophagy (Fig. 1D). First, ROS trigger an indirect mechanism whereby a pathological burst of ROS within a dysfunctional mitochondrion can trigger opening of a mitochondrial permeability transition pore (mPTP) [62,63]. mPTP is a nonselective voltage-dependent channel that releases small solutes across the inner mitochondrial membrane (IMM) and collapses the electro-chemical gradient achieved by proton (H^+^) pumping by the electron transport chain [63]. Loss of mitochondrial membrane potential leads to the inhibition of mitochondrial protein import pores and thus promotes stabilisation and activation of PTEN-induced putative kinase 1 (PINK1) on the outer mitochondrial membrane [64]. Activity of the PINK1-Parkin-ubiquitin axis leads to dysfunctional organelle recognition by autophagy receptors and recruitment of the autophagic machinery [65].

Second, ROS released within the mitochondrial matrix promote oxidative modification of cardiolipins (CL), an abundant family of mitochondria-specific phospholipids located predominantly in the inner leaflet of the IMM. Within healthy mitochondria, CL functions to stabilise mitochondrial electron transport chain complex and supercomplex assembly and promote ETC activity (reviewed in Ref. [66]). However, increase in mitochondrial ROS release was shown to promote human phospholipid scramblase-3 (PLS3)-assisted CL translocation from the IMM to the outer leaflet of the outer mitochondrial membrane (OMM) [67]. Supported by computational modelling and experimental evidence, externalised CL was demonstrated to act as a receptor for mitophagy due to its interaction with the N-terminus of LC3 and selective recognition of dysfunctional organelles [67]. Although the specific stimuli and mechanisms of CL oxidation *in vivo* remain elusive, an extensive review by Yin and Zhu provides evidence for oxidative modification of CL by different ROS, including OH^•^ and H_2_O_2_, leading to the formation of numerous oxidation products with unknown physiological roles that require further characterisation [68].

Finally, the involvement of ROS in the initiation of a well-characterised hypoxia-induced mitophagy is a topic of much controversy. The canonical pathway is regulated by hypoxia-inducible factor-1 (HIF-1), a heterodimeric transcription factor that is stabilised in low oxygen conditions due to the loss of upstream degradation signalling [69]. Stabilised HIF-1, formed as a dimer of O_2_-sensitive HIF-1α and constitutive HIF-1β subunits, then binds to hypoxia response elements (HREs) in the promoter regions of noncoding RNAs and proteins that regulate autophagy, angiogenesis, cellular metabolism and apoptosis [70,71]. Two proteins that fall under the transcriptional regulation of HIF-1 are BNIP3 and NIX, noncanonical BCL-2 family proteins that interact with BCL-2 to release Beclin 1 from a BCL-2-Beclin 1 heterodimer and allow Beclin 1 association with the Class III PI(3)-kinase complex [61,70,72]. Moreover, BNIP3 and NIX contain a transmembrane glycine zipper domain that promotes their dimerisation and insertion into OMM [73,74]. Both proteins also physically interact with LC3 [75,76] and recruit the autophagic machinery to superfluous mitochondria, promote mitochondrial recycling and altogether decrease mitochondrial ROS production. Although initially described for their role in hypoxia, an increasing body of contradictory evidence addresses whether ROS can directly and indirectly promote HIF-1 stabilisation (discussed in Ref. [77]). Despite the described controversy in the topic of HIF-1 ROS-induced stabilisation on mitochondria, the literature agrees that exogenous addition of ROS or inhibition of superoxide dismutases promote HIF-1 stabilisation under normal oxygen conditions [77], which may be sufficient for mitochondrial removal by BNIP3 and NIX. However, more needs to be understood about the mechanism of HIF-1 stabilisation, and BNIP3 and NIX targeting specifically to ROS-producing organelles.

Peroxisomes are highly metabolic organelles that release ROS as a by-product of long-chain fatty acid β-oxidation. In contrast to mitochondria which transfer liberated electrons to the ETC, electron release during β-oxidation in peroxisomes results in uncontrolled O_2_ reduction to H_2_O_2_ [78]. In addition, several other mechanisms of O_2_^•−^ production were identified, including the activity of xanthine oxidase and systems on the peroxisomal membrane that use reduced nicotinamide adenine dinucleotide (NADH) and reduced phosphorylated NAD (NADPH) as electron donors (reviewed in Ref. [79]). Localised ROS release from peroxisomes triggers organelle recycling in an Ataxia-telangiectasia mutated (ATM) kinase-dependent manner. In addition to its role as a DNA damage sensor in the nucleus, ATM was also found to colocalise with the outer surface of the peroxisomal membrane via its interaction with peroxin 5 (PEX5), a peroxisome import receptor (Fig. 1E) [80]. Although the physiological role of ATM interaction with PEX5 in basal conditions is unknown, it was previously reported that ATM kinase activity is enhanced by an intermolecular Cys^2991^-dependent disulphide bond formation [81]. Consequently, exogenous H_2_O_2_ addition into culture media of a cellular model promoted ATM colocalisation with PEX5 and led to phosphorylation of PEX5 serine 141 (Ser^141^) residue that primed PEX5 for subsequent lysine 209 (Lys^209^) monoubiquitylation [80]. In turn, PEX5 monoubiquitylation was shown to recruit NBR1 (and p62) and, by extension, the autophagic machinery and thus stimulate pexophagy (Fig. 1E) [[82], [83], [84]]. In addition to its direct interaction with PEX5, H_2_O_2_-activated ATM also promoted a phosphorylation cascade of the ATM-tuberous sclerosis complex 2 (TSC2) mammalian target of rapamycin complex 1 (mTORC1) axis that repressed mTORC1 activity and stimulated autophagy [80]. Altogether, pexophagy is stimulated by ROS in a redox-sensitive ATM-dependent manner that promotes autophagy initiation downstream of mTORC1 inhibition and selective peroxisome targeting via PEX5 ubiquitylation.

## ROS and Autophagy in Neuronal Health

The central nervous system (CNS) is uniquely sensitive to oxidative stress due to its anatomical and functional features. The low rate of neuronal regeneration, the exceptionally high demand for energy, the high lipid content, the high concentration of NO and the low level of antioxidant defences result in an increased vulnerability of the brain to elevated ROS levels [85]. CNS vulnerability to age-related decline can result in a range of sporadic neurodegenerative disorders. This heterogeneous group of disorders greatly varies with regards to the age of onset, brain regions affected by pathology, symptoms and is likely to arise as a result of multiple and variable causes [86]. Molecular disruption in neurodegenerative disorders commonly includes increased oxidative stress, mitochondrial dysfunction, the presence of abnormal protein aggregates and aberrant proteostasis [87].

### ROS in neuronal health and neurodegeneration

ROS act as signal messengers in a healthy brain. Their generation and diffusion help propagate signalling cascades important for neuronal development and function [88]. Specifically, a burst of ROS was found to precede the establishment of neural polarity *in vitro* [89], synaptic structural plasticity in *Drosophila* [90], synaptic functional plasticity in rat hippocampus [91] and was implicated in neurogenesis in neural stem cells [92]. On the flip side, more is known about the deleterious role of reactive species. Increased levels of oxidative protein modification are often correlated with ageing and neurodegenerative disease pathology (reviewed in Ref. [93]). A positive correlation between age and cysteine oxidation levels was first reported from plasma of healthy volunteers [94]. In addition, cysteine modification by ROS/RNS species was linked to dysfunction of proteins implicated in various neurodegenerative disorders [93]. First, multiple substrates of S-nitrosylation seem to be oxidised in multiple neurodegenerative disorders including amyotrophic lateral sclerosis (ALS), Alzheimer's (AD), Huntington's (HD) and Parkinson's diseases (PD) and are perhaps indicative of increased oxidation levels rather than causative in disease (reviewed in Ref. [95]). These substrates include X-linked inhibitor of apoptosis protein (XIAP), protein disulphide isomerase (PDI) and glyceraldehyde-3-phosphade dehydrogenase (GAPDH) that function in cellular apoptotic, endoplasmic reticulum (ER)-stress and energy generation pathways, respectively.

Second, oxidative modifications can affect proteins demonstrated to participate in disease pathology. Oxidative PTMs of tau including intermolecular disulphide bond formation or Cys glutathionylation was found to promote formation of paired helical filaments that underlie the molecular structure of neurofibrillary tangles that were together with amyloid β (aβ) plaque deposition identified as pathological hallmarks of AD [96]. Redox proteomic analysis of AD patient brain samples also led to identification of a number of redox-regulated enzymes involved in glucose oxidation and energy metabolism pathways [97] the dysfunction of which was previously linked to AD (reviewed in Ref. [98]). Similarly, oxidative modification of wild-type superoxide dismutase 1 (SOD1) by sulfenic acid formation on Cys^111^ residue can result in SOD1 oligomer and fibril formation and thus induce apoptosis. This mechanism appears to be relevant to sporadic ALS as increased levels of sulfenic acid–modified SOD1 were detected in the cerebrospinal fluid of ALS patients [99].

Oxidative PTMs were also reported in proteins genetically linked to familial Parkinson's disease (PD) that is characterised by selective loss of dopaminergic neurons in the substantia nigra pars compacta region of the midbrain [100]. On a molecular level, the pathology of PD is multifactorial and includes formation of proteinaceous bodies rich in alpha-synuclein (α-syn), and mitochondrial dysfunction [100]. Evidence suggests that both molecular pathologies can be affected by ROS. PD onset and progression seem to correlate with depleted levels of a nonenzymatic antioxidant, glutathione, that come hand in hand with increased oxidative stress modification and dysfunction of proteins identified in familial forms of PD [93,101]. First, although α-syn contains no native cysteine residues, oxidation of α-syn methionine residues on interaction with dopamine (DA) was found to lead to formation of DA-α-syn adducts that precede toxic soluble oligomer formation [102]. Second, oxidative modification of Cys^106^ residue of DJ-1 to sulfinic acid [103], and downregulation of DJ-1 expression in response to oxidative stress [104] both led to aberrant α-syn aggregation. And finally, two oxidative modifications of Parkin, S-nitrosylation and sulfonylation were detected in PD [[105], [106], [107]]. Parkin S-nitrosylation on exposure to NO was independently shown to inhibit the E3 ubiquitin ligase activity in cell models [105,106], although this was preceded by a short-term increase in activity in one of the studies [105]. In addition, increased levels of Parkin S-nitrosylation were detected in brain samples from PD patients [105,106]. Similarly, Parkin sulphonation on cell exposure to exogenous H_2_O_2_ led to an initial stimulation of its E3-ubiquitin ligase activity, followed by a decrease, which authors hypothesise is due to excessive autoubiquitylation [107]. A decrease in Parkin activity due to chronic ROS exposure could thus lead to loss of mitochondrial recycling. These studies demonstrate that increased levels of oxidative stress could underlie both pathological hallmarks of PD and provide an explanation for the selective vulnerability of dopaminergic neurons to cell death. Importantly, they also highlight another candidate protein, Parkin, as a ROS-sensitive regulator of selective autophagy that might show a similar activity feedback loop that was reported in the study of ATG3 and ATG7 redox regulation [37].

### Autophagy in neuronal health and neurodegeneration

The autophagy pathway is spatially tuned in neuronal cells. Autophagosomes were found to form in neurites and most travel along the axonal microtubules to the neuronal soma to fuse with lysosomes [108]. On a physiological level, autophagy was demonstrated to support neurogenesis and neuronal development and protect cells from stress-induced loss of viability [108]. Studies of healthy ageing and familial neurodegenerative diseases highlight the need for functional autophagy in the aged brain. Owing to the high levels of ROS/RNS, yet relatively low level of antioxidants, the second line of antioxidant defence, including the proteasome- and autophagy-mediated substrate degradation, is crucial for redox status maintenance in the brain and prevention of age-related neurodegeneration [85]. Autophagy impairment has been reported from studies of postmortem tissue from patients suffering from neurodegenerative diseases [[109], [110], [111]] and in healthy aged human brains [112,113]. The link between functional autophagy and neuronal health is further supported by a series of studies focussing on key autophagy genes involved in the pathway initiation. These demonstrate that autophagy abolition alone is sufficient to drive protein aggregation, neuronal death and early-onset neurodegeneration (reviewed in more detail in Ref. [114]). In addition to the core autophagy proteins, dysfunction of or mutations in many proteins linked to neurodegeneration are now recognised for their direct (autophagosome formation, cargo recognition) or indirect (lysosome dysfunction) involvement in the autophagy pathway [114].

Alterations in autophagy flux in AD were first observed by electron microscopy imaging of AD brains [109] and later confirmed in a familial form of AD, where loss presenilin-1 function leads to insufficient lysosome acidification, an end-stage block to autophagy flux and deposition of amyloid plaques [115]. In addition, low expression levels of Beclin 1, a member of the class III PI(3)-kinase complex, were reported from AD patient brain tissue [110], which was also characterised by increased amyloid deposition in a mouse model. In addition, both proteins that are characterised in AD pathology, Aβ and tau, and aggregate in either plaques (Aβ) or tangles (tau), are substrates of autophagy [116,117]. Importantly, amyloid plaques were found to contain a high amount of zinc, iron and copper metals that can lead to aberrant ROS production [118] and result in oxidation of other cellular components. This sequence of events would support findings from a model of AD pathology [119], whereby disease progression is underpinned by age-related autophagy dysfunction that leads to amyloid deposition, ROS release and exacerbated tau pathology. However, the role of either hallmark of AD pathology or indeed their interaction, is still highly disputed and the sequence of molecular events requires further study [120].

HD is characterised by trinucleotide repeat expansion in the huntingtin gene that results in a toxic gain of function of the expressed protein and aberrant autophagy [121,122]. Similarly to Aβ and tau, huntingtin is a substrate of autophagy. In addition, huntingtin was shown to stimulate autophagy *in vitro* by potentially acting as a scaffold for selective cargo recognition (reviewed in Ref. [121]), although the relevance of the study to an *in vivo* scenario is unknown. Interestingly, a common V471A polymorphism of ATG7 was identified as a modifier of HD in heterozygous carriers in German and Italian populations [123,124]. However, the effect of the V471A mutation on ATG7 function in autophagy, or the redox-sensitive Cys^572^ residue implicated in autophagy modulation [37], remains unknown.

Familial forms of ALS, the most common form of motor neuron disease, are among others, linked to two selective autophagy receptors, p62 and optineurin involved in recognition and clearance of protein aggregates [125]. The nature of protein aggregation and an interdependence between potential dysfunction of selective cargo recognition and neuronal toxicity in ALS is currently unknown as, at least in p62 studies, disease-causing mutations have been identified in multiple regions of the protein and lack a clear link between the site of mutation and loss of function [126].

α-Syn mutation or aggregation in PD can affect autophagy flux in several ways. First, increased α-syn levels were sufficient to inhibit autophagy in cell culture and animal models including *Drosophila* and mouse [127] by interfering with ATG9 localisation to the site of autophagosome formation. In addition, excessive α-syn levels interfered with TFEB translocation to the nucleus and thus impaired expression of lysosomal and autophagy genes in a rat midbrain [128]. Moreover, changes in TFEB localisation were also detected in postmortem brain samples from PD patients [128]. The clearest link between autophagy impairment and PD comes from studies of PINK1-Parkin mediated mitophagy [64,65]. Loss of function of either protein leads to loss of mitochondrial recycling in response to depolarisation and autosomal recessive onset of PD. Altogether, loss of proteostasis that presents as aberrant protein aggregate formation and autophagy impairment is common to many familial and sporadic forms of neurodegenerative disease but the exact contribution of autophagy dysfunction to disease pathology is not completely understood.

### ROS-Autophagy axis in neurodegeneration

Although it is widely recognised that autophagy dysregulation and increased oxidative stress play a key role in ageing and underlie many inherited and spontaneous neurodegenerative diseases, the links between excessive ROS and autophagy dysfunction in the human brain remain elusive. The lack of understanding of the causative relationship in human neuronal health is underpinned by several confounding factors. Studies of human familial and sporadic disease are carried out on postmortem tissue, which can inform about the end state of disease but not about its progression and the causality of observed pathology in disease [129]. The use of animal models addressed some of the problems and enabled researchers to gain insight into pathological features and biology of disease by studying transgenic animals and to infer causality by tissue/cell-type specific mutant protein targeting [130,131]. However, the extent to which model animals reconstitute human disease pathology varies greatly and translation of research findings from the laboratory to the clinic remains challenging.

Moreover, the multifactorial and varied nature of dysfunction in sporadic disease combined with the lack of reliable biomarkers and the difficulty with obtaining data due to the inaccessibility of brain tissue complicate the effort of a patient-specific diagnosis and treatment tailoring. The conflicting results of crosstalk between ROS and autophagy complicate the issue on a molecular level. Although short bursts of ROS tested in cellular and animal models increase autophagy flux [16,22,23,32], the effects of chronic exposure to elevated ROS levels, in some cases combined with a decreased capacity of the antioxidant system, are unclear. It is currently widely accepted that ROS activate autophagy as a cytoprotective feedback mechanism that restores cellular homeostasis. However, we can only hypothesise whether chronic ROS formation in the context of inefficient autophagic flux would have the same, or an entirely opposite effect.

And finally, no disease-causing genetic mutations of cysteine residues in autophagy proteins susceptible to oxidative modification have been reported. The two notable exceptions come from proteins involved in selective cargo degradation, including identification of six disease-causing mutations of redox-sensitive cysteines in Parkin [107] and our study of the K102E mutation in p62 that is relevant to a subset of sporadic ALS cases [47]. In the *PARK2* gene, disease-causing mutations in Cys^212^, Cys^268^, Cys^289^ and Cys^441^ made Parkin more susceptible to aggregation, often into a single mass, when compared to wild-type or other disease-causing mutations [132]. In this case, loss of mutated Parkin activity and disease onset occurs likely due to its sequestration in protein aggregates rather than loss of redox-sensing activity. By contrast, we have demonstrated that a missense mutation in the *SQSTM1* gene that is causative in sporadic form of ALS, the K102E mutation, impaired the formation of intermolecular disulphide bond formation and, by extension, reduced the redox sensitivity of p62 [47]. As new discoveries of redox-sensitive proteins involved in the autophagy pathway are reported [37,123,124], it will be interesting to see whether other common polymorphisms in autophagy machinery are identified as redox-sensitive disease modifiers and whether development of targeted therapy becomes a possibility.

## Concluding Remarks and Perspectives

Multiple lines of evidence from patient tissue and animal models implicate autophagy dysfunction in the onset and progression of neurodegenerative diseases. Similarly, while low levels of ROS release may be beneficial as messengers mediating cellular development and adaptation to stress, chronic exposure to elevated ROS levels and the resulting oxidative modification of cysteine residues can interfere with mitochondrial function, neuronal synapse and cell survival. The interplay between autophagy and ROS in healthy cells seems to be beneficial. Short bursts of ROS mediate autophagy activation at the level of transcription, protein enzymatic activity and substrate recognition and thus create an important link between the need for degradation of oxidised cellular substrates and the efficiency of the autophagic pathway. Current evidence also suggests the presence of a self-regulatory system by which ROS-mediated inactivation of ATG3 and ATG7 proteins, Parkin autoubiquitylation and increased degradation of p62 via autophagy prevent excessive cellular catabolism in response to ROS signals. However, evidence from ageing and neurodegenerative models indicates that this regulatory link is broken by either decreased cellular detoxification due to loss of autophagic flux, or elevated ROS formation that overwhelms cellular cytoprotection and leads to the loss of cell viability. Although the evidence of autophagy redox-sensitive protein involvement in neuronal disease pathology is lacking, we hypothesise that chronic ROS exposure highjacks the self-regulatory autophagy systems, thus promoting a sustained block in autophagy flux and promotes establishment of a vicious cycle of toxicity and damage.
